# CT and chest radiography in evaluation of mechanical circulatory support devices for acute heart failure

**DOI:** 10.1186/s13244-023-01469-8

**Published:** 2023-07-16

**Authors:** Abhishek Chaturvedi, Yonatan Rotman, Timothy Hoang, Greg Jew, Aniruddh Mandalapu, Craig Narins

**Affiliations:** 1 Department of Imaging Science, Cardiothoracic Imaging, University of Rochester Medical Center, 601 Elmwood Ave, Rochester, NY 14562 USA; 2 Department of Medicine, Interventional Cardiology, University of Rochester Medical Center, Rochester, NY USA

**Keywords:** Intra-aortic balloon pump (IABP), Impella pump, Impella RP, Protek Duo, Tandem Heart

## Abstract

**Abstract:**

Acute heart failure and cardiogenic shock are a major cause of morbidity and mortality in patients who have had recent cardiac surgery, myocardial infarct or pulmonary hypertension. The use of percutaneous mechanical circulatory support (MCS) devices before organ failure occurs can improve outcomes in these patients. Imaging plays a key role in identifying appropriate positioning of MCS devices for supporting ventricle function. These devices can be used for left ventricle, right ventricle or biventricular support. Fluoroscopy, angiography and echocardiography are used for implanting these devices. Radiographs and CT can identify both intra- and extra-cardiac complications. The cardiothoracic imager will see increasing use of these devices and familiarity with their normal appearance and complications is important.

**Critical relevance statement:**

Chest radiographs and CT are useful for assessing the position of the mechanical cardiac support device used for treatment of acute heart failure. CT can identify cardiac and extra-cardiac complications associated with these devices.

**Graphical abstract:**

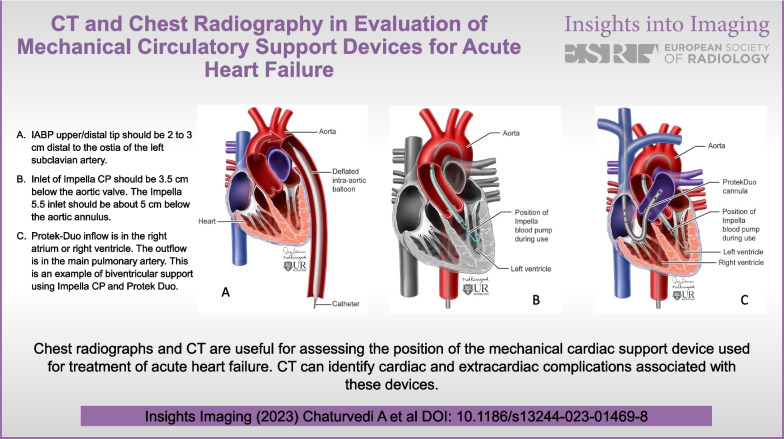

**Key points:**

- IABP upper/distal marker should be 2–3 cm distal to the ostia of the left subclavian artery.
- Inlet of Impella CP should be 3.5 cm below the aortic valve.
- The Impella 5.5 does not have a pigtail portion. The inlet should be about 5 cm below the aortic annulus.
- Impella RP inlet port should be in the right atrium or inferior vena cava, the pigtail portion should be positioned in the main pulmonary artery.
- Protek Duo inflow is in the right atrium or right ventricle. The outflow is in the main pulmonary artery.

**Supplementary Information:**

The online version contains supplementary material available at 10.1186/s13244-023-01469-8.

## Introduction

Acute heart failure (AHF) is characterized by a decrease in cardiac function, shock and a very high mortality of up to 80% [1]. The hemodynamic criteria for AHF includes sustained hypotension (systolic blood pressure < 90 mm Hg for more than 30 min or the need for supportive measures to maintain this pressure) and reduced cardiac index (less than 2.2 L/min/m^2^) with an elevated pulmonary capillary wedge pressure (15 mmHg) [2]. In the National Health and Nutrition Examination Survey data, heart failure (HF) affects more than 6 million individuals greater than 20 years old in the US [3]. There are about 250,000–300,000 patients younger than 75 who suffer from advanced systolic HF (defined as NYHA class IIIb–IV) [2]. The Interagency Registry for Mechanically Assisted Circulation (INTERMACS) classification system risk stratifies patients with advanced HF to better define prognosis and urgency of intervention [4]. Mechanical support of circulation is becoming important therapy when heart failure is refractory to medical therapy [5]. These devices are used as means of supporting patients with AHF and circulatory shock from myocardial infarction, and those undergoing percutaneous or surgical revascularization [6]. The goal of treatment is to restore systemic perfusion and prevent heart and organ failure.

**Fig. 1:**
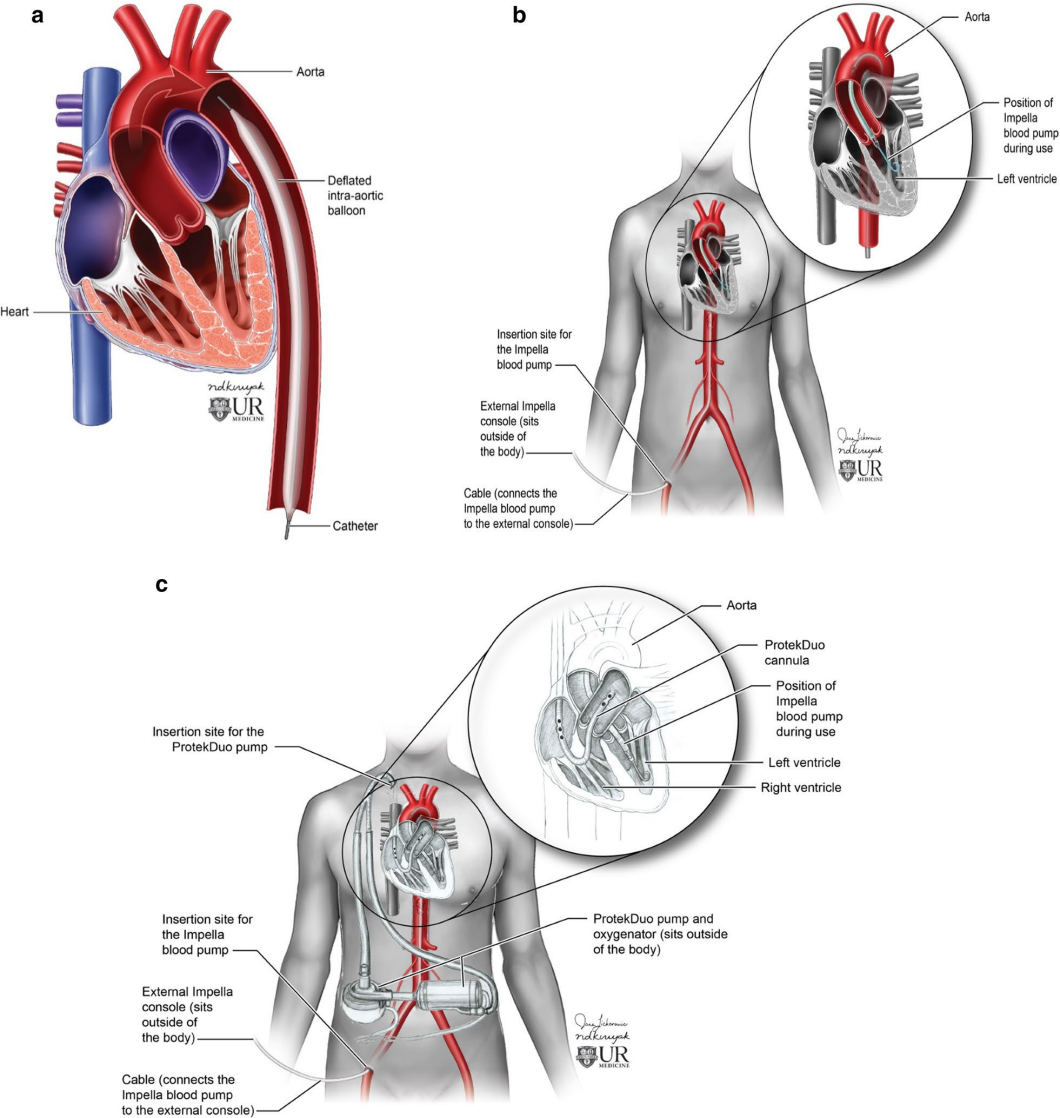
**a** Illustration demonstrates the position of the intra-aortic balloon pump placed from the femoral artery. **b** Illustration demonstrates the normal position of the Impella CP LV support system. The device is implanted from the femoral artery. Impella pump can also be implanted through the axillary artery or aorta. **c** Illustration demonstrates the normal position of the Protek Duo in the main pulmonary artery and the Impella CP LV support system. In this illustration, we see use of two separate devices for biventricular support

Devices for AHF: Mechanical circulatory support (MCS) devices used for AHF provide temporary circulatory support for a short time (Table 1). These devices include the intra-aortic balloon pump (IAPB), and Impella pump for the left ventricle (LV) support (Fig. 1A, B). Right ventricle (RV) support can be direct or indirect [7]. Direct RV MCS devices include Impella RP, Protek Duo (Fig. 1C) and Tandem Heart. Indirect RV support devices include peripheral or central veno-arterial extracorporeal membrane oxygenation (VA ECMO). In some instances, patients may require biventricular support (Fig. 1C). In these cases, both LV and RV MCS devices are placed for hemodynamic support.

**Table 1:** The commonly used mechanical circulatory support device for acute heart failure

Device	Mechanism	Type of flow	Normal location	Comments
IABP	Counter pulsation	Pulsatile	Upper marker: proximal to ostia of subclavian artery Lower marker: above celiac trunk [9–11]	Contraindications: Aortic insufficiency, aortic dissection or aneurysm
Impella	Axial flow	Continuous	Impella CP: Inflow: 3.5 cm below aortic valve Impella 5.5: Inflow port 5 cm from the aortic valve plane [14–16, 30, 31] Outflow in ascending aorta	Contraindication: aortic insufficiency, aortic dissection or aneurysm, severe PVD, VSD. Mechanical aortic valve, RV failure
Impella RP	Axial flow	Continuous	Inflow: right atrium or superior vena cava Outflow: main pulmonary artery or left pulmonary artery [7, 21, 22, 25]	Outflow should be positioned in the main pulmonary artery
Protek Duo	Centrifugal	Continuous	Inflow: superior vena cava to right atrium Outflow: main pulmonary artery or left pulmonary artery [7, 26–28]	Outflow should be positioned in the main pulmonary artery
Tandem Heart	Centrifugal	continuous	Inflow: right atrium or superior vena cava Outflow: main pulmonary artery or left pulmonary artery	Used with a central VA ECMO device or Protek Duo

Complications associated with the MCS devices include malposition, cardiovascular injury and hematoma. Thrombus can form in the access vessel along the cannulae and there can be distal emboli (Table 2).

**Table 2:** Imaging findings of mechanical circulatory support device complications

Complication	CXR	Echo/US	CT
Malposition	Markers not at expected landmarks Change in position compared to prior imaging	Markers not at expected landmarks Change in position compared to prior imaging	Markers not at expected landmarks Change in position compared to prior imaging
Hematoma	New mediastinal widening or pleural parenchymal opacities	Hemopericardium with/without tamponade	High attenuation fluid collection. Active contrast extravasation on contrast enhanced CT
Cardiovascular perforation	Tip beyond the cardio mediastinal contour	Inability to dislodge the device	Tip in the mediastinum with associated hemopericardium or hemothorax
Thrombus	–	Echogenic lesion abutting the device	Acute thrombus can be hyperdense to blood pool on non-contrast CT Chronic Thrombus can have calcifications Thrombus is hypodense to myocardium on contrast enhanced CT
Embolus	–	Decreased/absent peripheral arterial flow	Signs of infarcts/ischemia due to distal emboli

Echocardiography remains the primary modality to evaluate cardiac function, guide placement of these devices and allow for access during cardiac complications. Chest radiographs (CXR) can play an important role in assessing the location of the cannulae, and for assessing cardiopulmonary status. Ultrasound with Doppler is useful to evaluate access vessel hematoma, thrombus or pseudoaneurysms.

The MCS devices are placed emergently under echocardiography or fluoroscopic guidance. These devices can get displaced during patient transfer. A chest radiograph is used for assessment after the patient is transfered to  the intensive care unit. Computed tomography (CT) is an important imaging modality to assess intra- and extra-cardiac complications (Table 3). When pre-implantation CT is available, it can be a useful adjunct to identify possible contraindications such as aortic aneurysms, dissections, cardiac thrombus, etc. MRI is contraindicated in patients with MCS devices.

**Table 3:** CT for evaluating patients with mechanical circulatory support devices

Type of scan	Indications	Comments
Non-contrast CT	Preoperative evaluation of lungs and mediastinum	Aortic intimal calcification Chest wall fluid collections, hematoma
Contrast enhanced CT	Pre- and postoperative evaluation for aorta, mediastinum and cardiac chambers	Aortic ectasia, aneurysm, cardiac thrombus, thoracic and abdominal embolic phenomena, active bleeding
Prospectively ECG-gated CTA	Pre- and postoperative evaluation of CABG grafts, thrombus, hematoma	Postoperative evaluation for MCS-associated thrombus, hematoma
Retrospectively ECG-gated CTA	Evaluation of native valves, cardiac function	Aortic and mitral valve and other interventions after LVAD such as transcatheter heart valve replacement

In this pictorial essay, we describe the expected normal appearance of the most commonly used MCS devices for AHF with the primary focus on radiography and CT. This work also describes the imaging findings of the commonly associated complications.

## Intra-aortic balloon pump

### Indication and mechanism

The intra-aortic balloon pump (IABP) is an internal counter-pulsation circulatory support device [8]. The balloon inflates during diastole augmenting coronary perfusion (after the aortic valve closure, triggered by the systemic arterial waveform or the T–P interval on ECG). The balloon deflates before the aortic valve opening at the onset of systole, correlating to the QRS–T interval. In atrial fibrillation, R wave triggers the deflation. This counter-pulsation augments the intrinsic Windkessel effect (potential energy stored in the aortic root during systole which is converted to kinetic energy with the elastic recoil of the aortic root) [6]. This reduces resistance to left ventricular ejection (afterload), cardiac work and myocardial oxygen consumption. With IABP assistance, there is a decline in systolic pressure by up to 10%, which indicates appropriate systolic unloading and afterload reduction [8]. Diastolic pressure can decrease by up to 30% and indicates systolic unloading.

The IABP pneumatic system has four components: a console, monitor for ECG and blood pressure, a valve and a source for gas [8]. The console delivers a specific volume of gas (helium or carbon dioxide) into a balloon at a predetermined time interval followed by retrieval of the gas. IABPs are implanted by transfemoral or transaxillary approach using a 7.5 or 8F (F = French) sheath. The IABP is mounted on a catheter and advanced into the descending aorta. The upper or distal tip of the IABP is radiopaque and should be positioned 2–3 cm distal to the ostia of the left subclavian artery.

### Normal imaging appearance

**Fig. 2:**
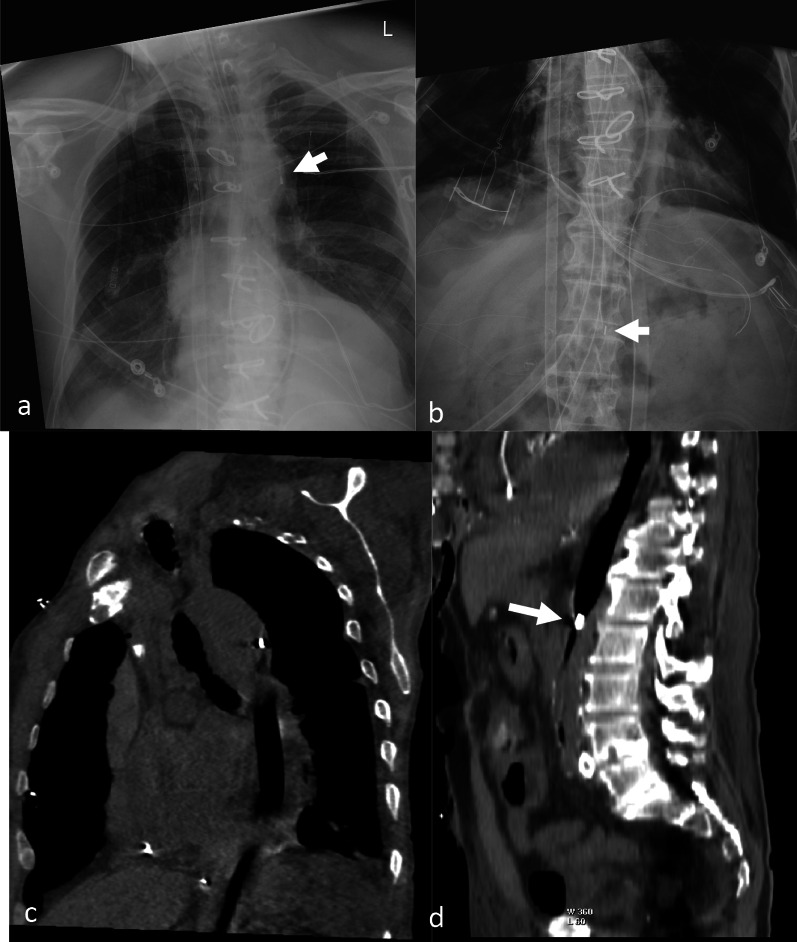
Intra-aortic balloon pump (IABP). Chest AP radiograph (**a**) demonstrating the upper radiopaque marker (white arrow) projecting over the aortic knob superior to the carina. This is the expected position based on radiography. Abdomen AP radiograph (**b**) in the same patient demonstrates the IAPB with the inferior radiopaque marker (white arrow) projecting over the L1-L2 Intervertebral space, which is low. Oblique coronal CT (**c**) demonstrates the IAPB in the descending aorta. The radiopaque marker is visible within the descending aorta about 5 cm from the origin of left subclavian artery. Sagittal CT abdomen image (**d**) demonstrates the low position of the IABP with its inferior radiopaque marker (black marker) below the level of the diaphragm at the level of the L1–L2 Intervertebral space. This can be seen when there is anatomic-to-balloon length mismatch

On anteroposterior chest radiograph, the correct position of the femoral IABP is defined as the location of the radiopaque upper marker 1 cm below the top of the aortic arch and at or above the level of the carina [9] (Fig. 2A). On CT, Rastan et al. described the balloon tip to be positioned 10–40 mm distal to the origin of the left subclavian artery (10 mm margin each to recommended distance of 20–30 mm) [10]. IABP from some vendors also has a lower or proximal radiopaque marker [11]. The ideal position of the lower marker has been described by different authors as, at the celiac trunk [10] or above the renal arteries [8]. The celiac trunk is at the level of T12 [12] and the renal arteries originate at a level over the lower third of the first lumbar vertebra and the lower border of the second lumbar vertebra [11, 13]. At our institution, the IABP is considered low if the lower marker of the balloon is below the L1 vertebra (Fig. 2A–D). IABP are also placed via the axillary artery. In these, the lower marker should be above the celiac trunk and the upper marker should not be in the subclavian artery.

The IABP size used is determined by the patient’s sex, height and age. The ideal balloon length should be less than the distance from the left subclavian artery to the celiac artery origin. The inflated diameter should be 90–95% of the diameter of the descending aorta. The most commonly used IABP in the adult patients has a 40 cc balloon, with membrane length (non-tapered section plus tapered ends) and inflated diameter varying between different manufacturers from 22 to 27.5 cm and 15–18 mm respectively [8] (Table 4).

**Table 4:** Size of the intra-aortic balloon pump (balloons placed through 7–9 F sheaths)

Balloon volume	Balloon membrane length (mm)	Inflated diameter (mm)
30 cc	230	13.9
40 cc	260	15
50 cc	262	17

In a study of 63 patients on IABP, Rastan et al. compared the transverse aortic diameter at the diaphragmatic level and the inflated diameter of each balloon on CT [10]. They suggested a residual lumen of ≥ 5 mm at the level of the diaphragm to be flow relevant. In this study, they found that the proximal balloon marker position was correct in 96.8% of patients when assessed by radiography but only appropriate in 38.1% based on CT [10]. This was due to incorrect balloon position and an anatomic-to-balloon length mismatch.

### Complications

**Fig. 3:**
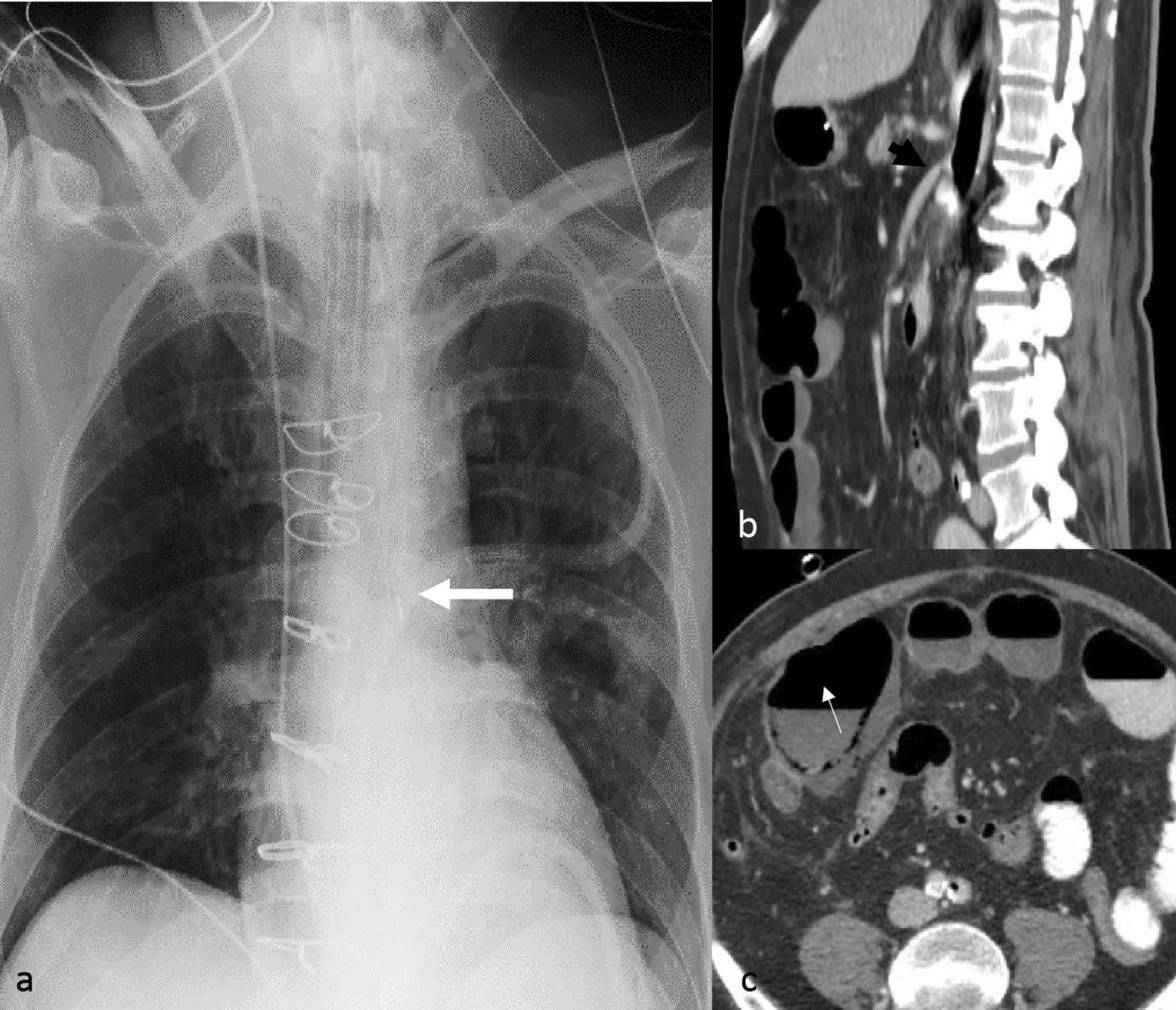
Complication from low IABP. AP chest radiograph (**a**) of a patient with an intra-aortic balloon pump. The upper radiopaque marker is below the carina (white arrow) which suggests a low position. Sagittal image (**b**) of a contrast enhanced chest and abdomen CT at the level of the celiac trunk and superior mesenteric artery (SMA) origin demonstrates high grade obstruction of the SMA and celiac trunk ostia by the distended IABP. Axial CT image (**c**) identifies complication from hypoperfusion: pneumatosis and contained perforation of the cecum (white arrow), due to the hypoperfusion from the high grade celiac trunk and SMA ostial occlusion. (IABP—Intra-aortic balloon pump)

**Fig. 4:**
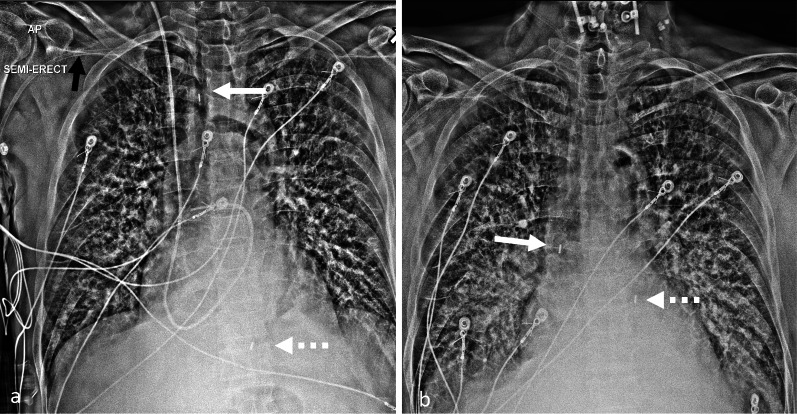
Malposition of axillary IABP. AP chest radiograph with contrast limited adaptive histone equalization (CLAHE) filter demonstrating an axillary approach IABP (**a**, **b**). The IABP is malpositioned with the upper marker in the brachiocephalic artery (white arrow on image **a**). The black arrow indicates the location of the sheath. The inflated balloon is seen in the aortic arch and upper descending aorta. The inferior marker is within the descending aorta (dashed arrow). **b** AP chest radiograph after attempted advancement of the IABP, the superior marker is malpositioned and is now in the ascending aorta (white arrow). The inferior marker is in the descending aorta (dashed arrow). (IABP = Intra-aortic balloon pump, CLAHE = Contrast limited adaptive histone equalization)

Complications associated with the IABP can be identified on imaging include malposition (Fig. 1), visceral arterial compromise (Fig. 3, Additional file 1: Movie S1) and cardiovascular injury. An uncommon complication seen with axillary approach IABP is malposition into the ascending aorta (Fig. 4). This can be due to difficulty maneuvering the device into the descending aorta.

## Impella LV support system

### Indication and mechanism

**Fig. 5:**
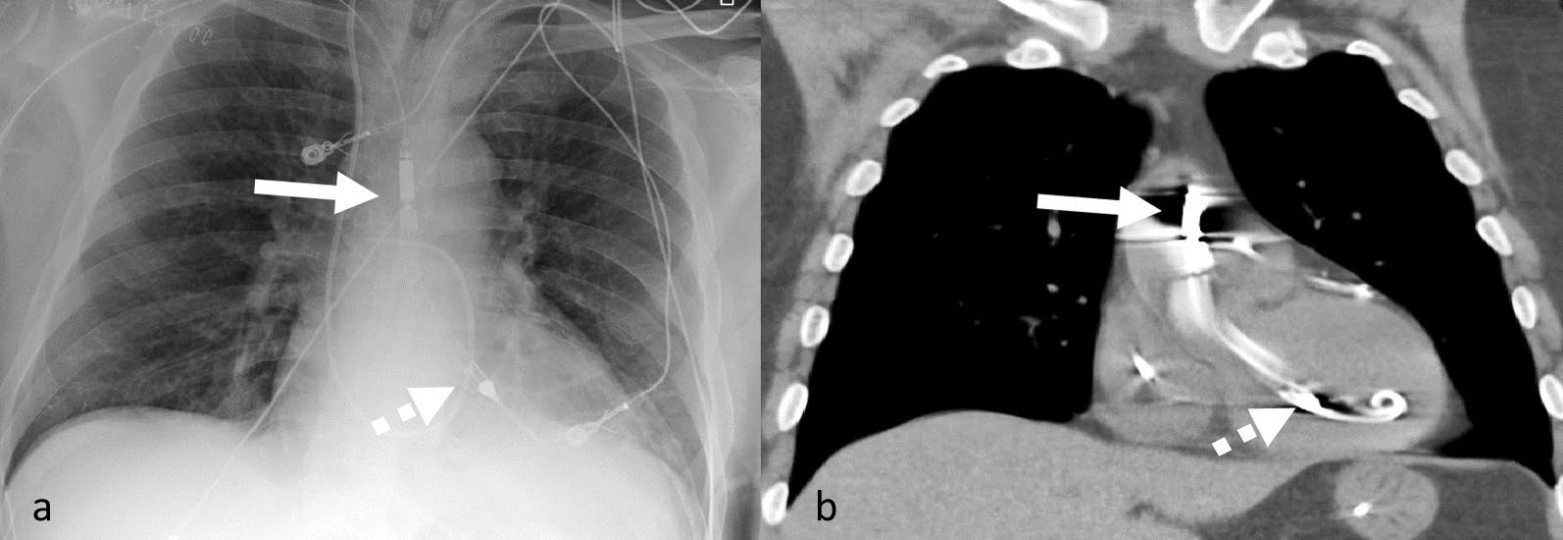
AP chest radiograph (**a**) and coronal CT (**b**) demonstrating a correctly positioned transaxillary Impella device. The outlet port is within the ascending aorta (white arrow) and the inlet port is projecting over the left ventricle (dashed arrow). The inlet of Impella CP should be 3.5 cm below the aortic annulus. The outlet should be in the ascending aorta. The pigtail portion is angled toward the LV apex away from the ventricle wall without mitral obstruction. (LV—left ventricle)

The Impella device (Abiomed Inc., MA, USA) is a catheter-based miniaturized ventricular assist device that pumps blood from left ventricle (LV) into ascending aorta [14]. This continuous flow device draws blood from the inlet port in the LV and expels it into the ascending aorta via the outlet port (Figs. 1B, C, 5). The US federal drug authority (FDA) has authorized the emergency use of the Impella LV support systems for temporary (≤ 4 days for Impella 2.5, Impella CP and Impella CP with SmartAssist; and ≤ 14 days for Impella 5.0 and Impella 5.5 with SmartAssist) LV unloading and support [15]. There are two Impella devices primarily in use for LV support: Impella CP, which is placed percutaneously and provides flow up to 4.4 L/min, and Impella 5.5, which provides flow up to 6 L/min and requires surgical implantation via an a 10-mm vascular graft sewn to either the axillary artery or the ascending aorta. The Impella catheter connects to the console, which displays rotations per minute, flow parameters, aortic and LV pressures. Use of the Impella device can be associated with higher rates of adverse events and costs [16]. In the PROTECT-II study (Protect II, A Prospective, Multicenter Randomized Controlled Trial) 452 patients undergoing high-risk PCI were randomly assigned to IABP or Impella 2.5. They found no difference in 30 or 90-day adverse cardiovascular events but did observe lower adverse events at 90 days in the Impella 2.5 arm [16, 17].

### Normal imaging appearance

The Impella CP is implanted using fluoroscopy and echocardiography guidance through the transfemoral or transaxillary approach using a 14F sheath. The distal pigtail portion of the Impella CP should be angled toward the LV apex, away from the mitral valve. The inlet port should be about 3.5 cm below the aortic valve and the outlet port should be in the ascending aorta. The inlet should be in the middle of the left ventricle, with the distal tip of the Impella pump oriented toward the apex of the left ventricle (Fig. 5) [18]. The Impella 5.5 does not have a pigtail portion. The inlet portion of the 5.5 should be about 5 cm below the aortic annulus [19]. The outlet port of all Impella pump should be in the ascending aorta [14]. For aortic insertion, the access is obtained about 7 cm above the aortic annulus.

### Complications

**Fig. 6:**
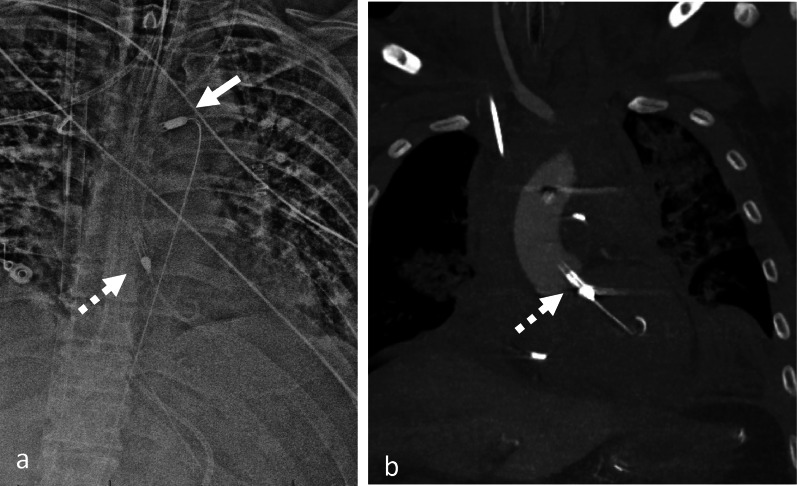
Malposition of Impella pump. AP Chest radiograph with CLAHE filter (**a**) with an Impella CP device with the outlet port in the aortic arch (white arrow) and the inlet port at the level of the aortic valve (dashed arrow). Contrast enhanced CT coronal image (**b**) at the level of the LVOT demonstrates high/proximal placement of Impella device with the blood inlet port at the level of the aortic valve. Contrast is seen only in the aorta but not in the left ventricle. This is due to backfilling from the VA ECMO cannula in the femoral artery. (CLAHE—contrast limited adaptive histogram equalization, LVOT—left ventricle outflow tract, VA ECMO—veno-arterial extracorporeal membrane oxygenation)

**Fig. 7:**
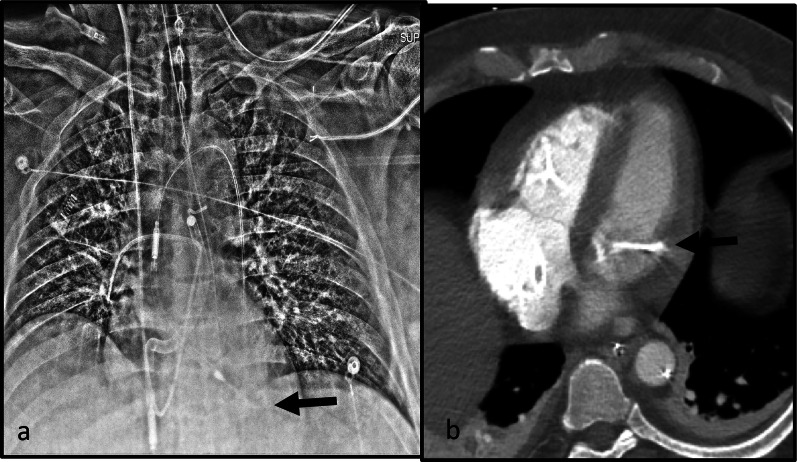
Malposition of Impella device. AP Chest radiograph with CLAHE filter (**a**) demonstrates a left ventricle Impella device with its tip projecting over the left ventricle (black arrow). Axial contrast enhanced CT (**b**) demonstrating the pigtail portion of the Impella device is entangled within the papillary muscle apparatus (black arrow). This finding is not detected on radiograph but can be identified on a CT. (CLAHE—contrast limited adaptive histogram equalization)

**Fig. 8:**
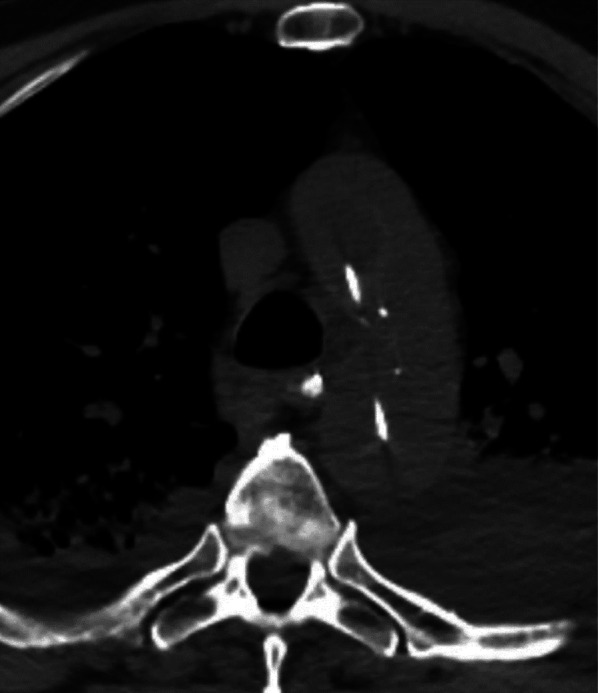
Complication of Impella device. Axial non-contrast CT in a patient on transfemoral Impella device demonstrates medial displacement of the intimal calcification. This is consistent with an acute aortic dissection

**Fig. 9:**
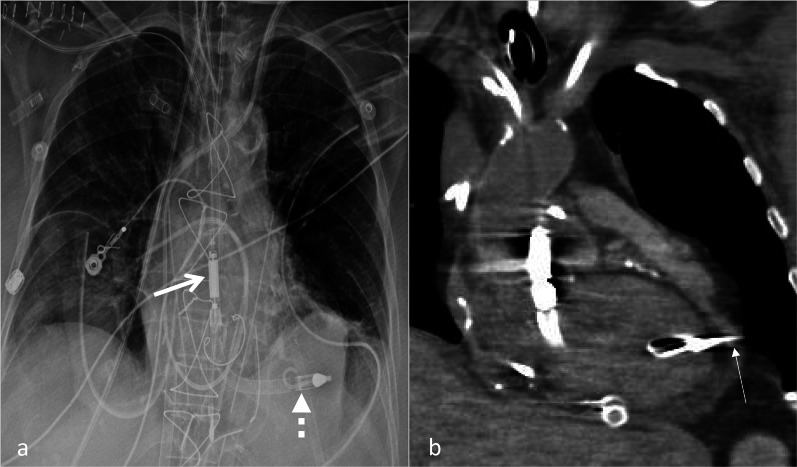
Complication from Impella. AP Chest radiograph with a left ventricle Impella device (**a**). The inlet port of the Impella is not in expected location on this radiograph, outlet port is much below the carina (white arrow) and the pigtail portion is coiled in the left ventricle cavity (white dashed arrow). The pigtail portioned is coiled, this is a sign of malposition. This can indicate entrapment within the papillary muscle apparatus. Non-contrast coronal CT at the level of the LVOT (**b**) confirms low positioned of the Impella device. The tip of the device has perforated the left ventricular wall (white arrow) and high density fluid can be seen in the pericardium, compatible with hemopericardium. (LVOT—Left ventricle outflow tract)

The common complications seen with the Impella pump include malposition (Figs. 6, 7) and vascular injury (Fig. 8). Rare complications include LV perforation (Fig. 9, Additional file 2: Movie S2).

## MCS for RV failure

MCS devices for RV failure unload the RV and decongest vital organs improving their function. RV failure treatment options include indirect RV bypass in the form of veno-arterial extracorporeal membrane oxygenation (VA ECMO) or direct RV bypass. Direct RV bypass devices are: Impella RP (axial flow), Protek Duo and Tandem Heart (Centrifugal flow). There are excellent publications on the VA ECMO device. An oxygenator can be spliced between the inflow and return cannula of Protek Duo and Tandem Heart [20].

## Impella RP

### Indication and mechanism

Impella RP (Abiomed Inc., MA, USA) is a micro-axial flow percutaneous MCS device, positioned from a single femoral venous access. It traverses the tricuspid and pulmonary valves. The outlet is positioned above the pulmonary valve in the main or the left pulmonary artery [21, 22]. Impella RP can deliver flow up to 4 L/min. This device cannot be used to oxygenate blood.

In the RECOVER RIGHT Trial, the central venous pressure and cardiac index improved after Impella RP implantation. This allowed weaning of inotrope and vasopressor support [23]. A recent prospective study at 14 US institutions in patients with life-threatening RV failure demonstrated rapid reversal of shock and favorable survival after Impella RP support [24].

### Normal imaging appearance

**Fig. 10:**
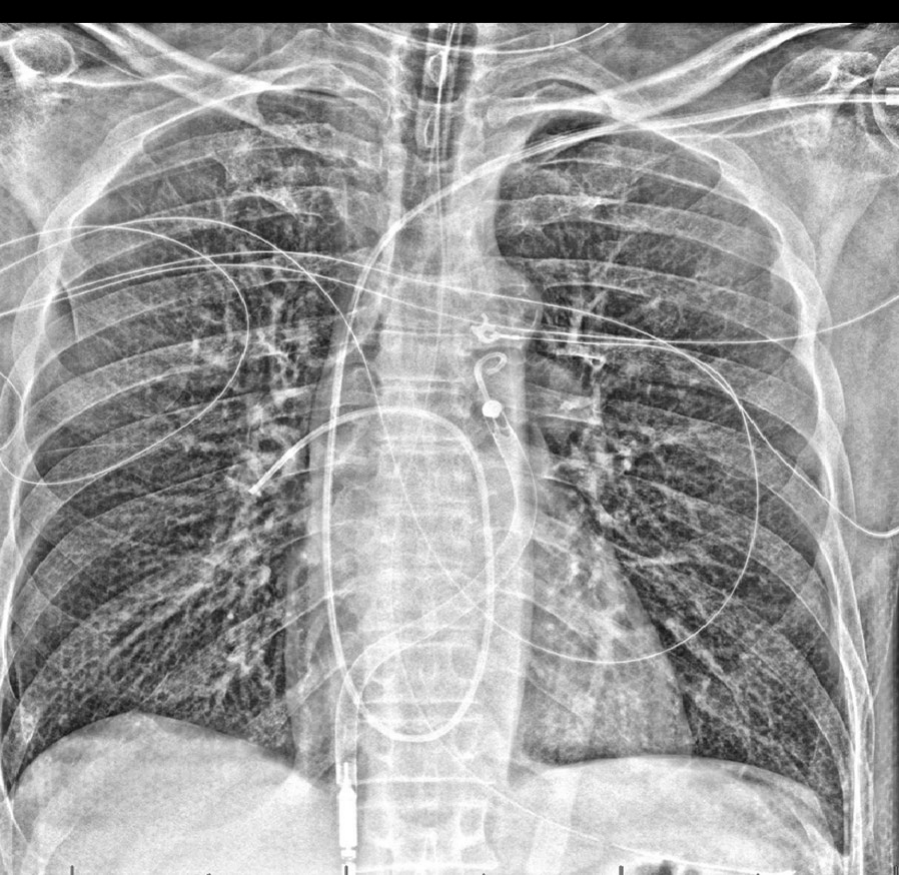
Chest radiograph with CLAHE filter in a patient with an Impella RP for right ventricle support. The pigtail portion in the outflow is in the main pulmonary artery. The inlet is in the IVC (white arrow). (CLAHE—contrast limited adaptive histogram equalization, IVC–inferior vena cava)

**Fig. 11:**
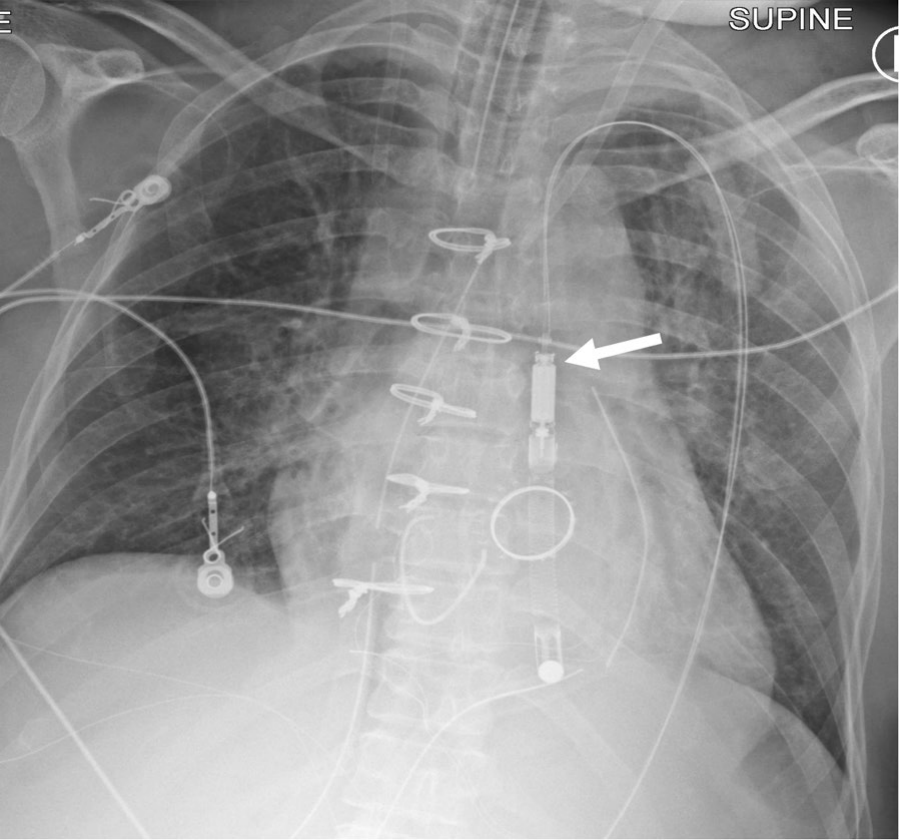
Chest radiograph demonstrating a right-sided placement of a LV Impella 5.5. This device was placed intraoperatively through a pulmonary artery graft. The inflow is located in the right ventricle and the outflow is located in the main pulmonary artery (white arrow) through the graft. The inlet and outlet directions can be changed depending on need to optimize flow. *Note*. There is no pigtail portion in the Impella 5.5 which helps differentiate this device. (LV—Left ventricle)

This device uses a 22 F impeller mounted onto an 11 F catheter. The inlet port should be in the right atrium or inferior vena cava, the pigtail portion should be positioned in the main pulmonary artery (Fig. 10). When Impella RP is not available, the Impella CP  or 5.5 have been used with reversal of flow direction (Fig. 11).

### Complications

Complications associated with Impella RP include ventricular arrhythmias, valvular regurgitation or progression of RV failure which can occur if the pump falls back into the RV [25].

## Protek Duo

### Indication and mechanism

**Fig. 12:**
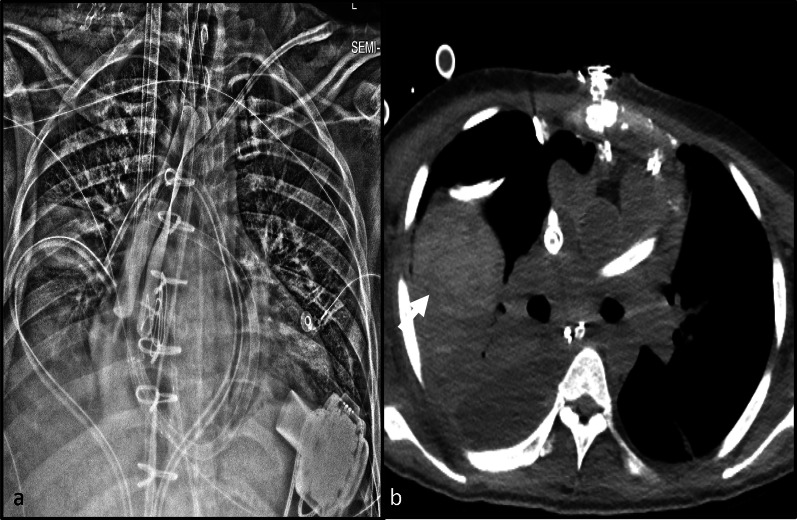
Protek Duo for right ventricle support. AP Chest radiograph with CLAHE filter after Protek Duo placement (**a**) demonstrating right basilar and mid-lung opacities. The outlet cannula portion should be in right ventricle and the inner portion within the pulmonary artery. In this case, the outlet cannula tip is within the right pulmonary artery. This can cause asymmetric perfusion and edema. Axial CT (**b**) in mediastinal window demonstrates asymmetric pulmonary opacities, right pleural effusion and a hemothorax (white arrow). (CLAHE—contrast limited adaptive histogram equalization)

The Protek Duo is an FDA approved, percutaneously placed, dual lumen veno-venous catheter system used for right ventricular bypass (Fig. 12) [26, 27]. It is placed over a guide wire (Seldinger technique) under fluoroscopy in the right internal jugular vein [28]. The outer inflow cannula has multiple vents. The inner return cannula tip also has multiple fenestrations. This cannula system is typically used with the Tandem Heart or CentriMag pump. The main advantage to using this cannula system is that only one internal jugular vein cannulation is necessary. Cannulation of the femoral vein is not necessary which may preserve patient mobility or use with femoral access is otherwise contraindicated. This is implanted after a 7 F sheath is placed and the internal jugular vein is serially dilated to allow passage of the 29 or 31 F Protek Dual Lumen Catheter [7].

### Normal imaging appearance

The outer inflow cannula has multiple vents and is positioned from the superior vena cava into the right atrium. The inner return cannula has multiple fenestrations and is positioned in the main pulmonary artery [7].

### Complications

Complications associated with Protek Duo include malposition into the right or left pulmonary artery with resultant asymmetric flow and edema. Rare complications include injury to the right ventricle wall (Additional file 3: Fig. S1) or pulmonary artery.

## Tandem Heart

### Indication and mechanism

The Tandem Heart pump is an extracorporeal centrifugal pump with percutaneously placed inflow and outflow cannulas. When used as an RVAD (Additional file 3: Figs. S2, S3), the inflow cannula is typically positioned in the right atrium with the outflow cannula in the pulmonary artery via the femoral veins. The pump can deliver flow rates up to 4.0 L/min at a maximum speed of 7500 rpm. Tandem Heart is a short-term mechanical support device designed and approved for use up to 30 days. The FDA-approved indications for the use of this device include cardiopulmonary failure or acute respiratory failure. For example, in cases of myocardial ischemia or volume overload after LVAD placement. The Tandem Heart can also be used in series with an oxygenator as part of an ECMO system. One of the disadvantages of using this device is that it typically requires cannulation of both femoral veins, limiting patient mobility. To ameliorate this issue, the Tandem Heart can be used with a Protek Duo, which requires only a single internal jugular cannulation.

In patients with RV failure after cardiac surgery, these cannulae can be placed directly into the right atrium and pulmonary artery through the open sternotomy.

## Biventricular mechanical circulatory support

RV failure after left ventricular assist devices implantation can be associated with high mortality [29]. In such scenarios, these percutaneous MCS devices can provide emergent support of the previously normally functioning right ventricle. In patients with LV failure on either acute or chronic MCS (implanted left ventricle support devices such as Heartmate II or HM3 for chronic heart failure: IABP or Impella for acute heart failure) with new onset RV failure, this support can be in the form of V-A/V-V ECMO, Impella RP or Protek Duo. In patients on MCS for RV failure with the new onset of LV failure, this can be in the form of Impella pump or IABP. The left ventricular sump/drainage cannula is placed from the aorta or by transvenous trans-septal puncture to decompress a dilated left ventricle. The tip of this catheter/cannula should be in the LV cavity. Recent clinical trials have demonstrated improved mortality and morbidity when these devices are used for RV or LV support. In some patients, a combination of these devices are being used for acute biventricular support.

Complications with these biventricular support devices include thromboembolism, mediastinal and distal bleeding (Additional file 3: Fig. S4), etc.

## Conclusion

There is an increase in the use of mechanical circulatory support devices for acute heart failure. Use of these devices significantly reduces mortality and morbidity when initiated before the onset of severe heart and systemic organ failure. The use of these devices can quickly stabilize patients with cardiogenic shock. Imaging plays a key role in identifying appropriate positioning of the MCS device. These devices are implanted under fluoroscopy and echocardiography. These can get displaced during patient transfer. The chest radiograph is routinely used for assessment after the patient is in the intensive care unit. CXR also serves as a baseline for monitoring cardiopulmonary status. CXR can also be the first imaging modality that identify complications associated with use of MCS for AHF. CT can identify both intra- and extra-cardiac complications on patients with mechanical circulatory support devices. CT is an important imaging modality in patients where echocardiographic images are suboptimal.

The cardiothoracic imager interpreting radiography, ultrasound and CT will likely see increased use of these devices. Familiarity with their normal appearance and complications is important. Chest radiographs and CT are useful for assessing the position of the mechanical cardiac support device used for treatment of acute heart failure. CT can identify cardiac and extracardiac complications associated with these devices.

## Supplementary Information

**Additional file 1.** ESM Video 1: Axial contrast enhanced CT of the chest abdomen and pelvis demonstrates low position of the intraoartic ballon pump.There is hypoperfusion and pneumatosis of the cecum. This is suggestive of ischemic colitis.

**Additional file 2.** ESM Video 2: Axial ECG gated CT of the thorax demonstrates malposition of the Impella pump. There is perforation of the left ventricle lateral wall. Pigtail portion of the Impella pump is present in the pericardium. There is a hemopericardium.

**Additional file 3.** Supplement figures.

## Data Availability

The information generated during and/or analyzed during the current study are available from the corresponding author on reasonable request.

## Abbreviations

AHF: Acute heart failure
CT: Computed tomography
CXR: Chest radiographs
eCPR: Extracorporeal cardiopulmonary resuscitation
IABP: Intra-aortic balloon pump
LV: Left ventricle
LVAD: Left ventricle assist device
MCS: Mechanical circulatory support
RV: Right ventricle
RVAD: Right ventricle assist device
VA ECMO: Veno-arterial extracorporeal membrane oxygenation
