# Supplementary material for: CT and chest radiography in evaluation of mechanical circulatory support devices for acute heart failure
Source: Insights Imaging. 2023 Jul 16;14:122. doi: 10.1186/s13244-023-01469-8 (PMC10350447; doi:10.1186/s13244-023-01469-8)
Supplement: Supplementary file 3 — Additional file 3. Supplement figures. [file 13244_2023_1469_MOESM3_ESM.pdf]

CT and Chest Radiography in Evaluation of Mechanical Circulatory Support Devices for Acute Heart Failure

ELECTRONIC SUPPLEMENTARY MATERIAL

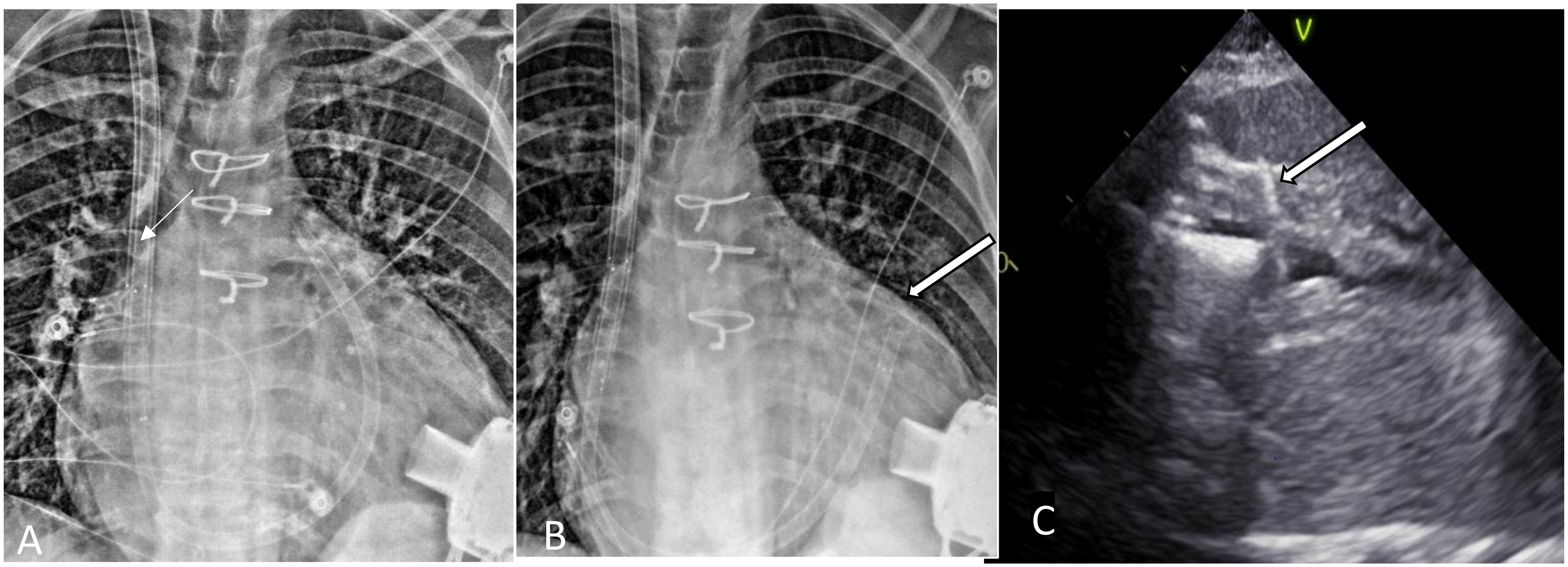

Supplemental Figure 1: Complication of Protek Duo a Patient with HeartMate3 LVAD and Protek Duo RVAD. Chest radiograph (A) shows appropriately positioned RVAD outflow cannula in the main PA (white arrow). Follow up radiograph (B) after 2 days shows the RVAD outflow cannula malposition with tip embedded in the RV wall (white arrow). Echocardiography with contrast (C) identifies bubbles extending from the right ventricle into the pericardium (white arrow) confirming the perforation with subsequent hemopericardium.

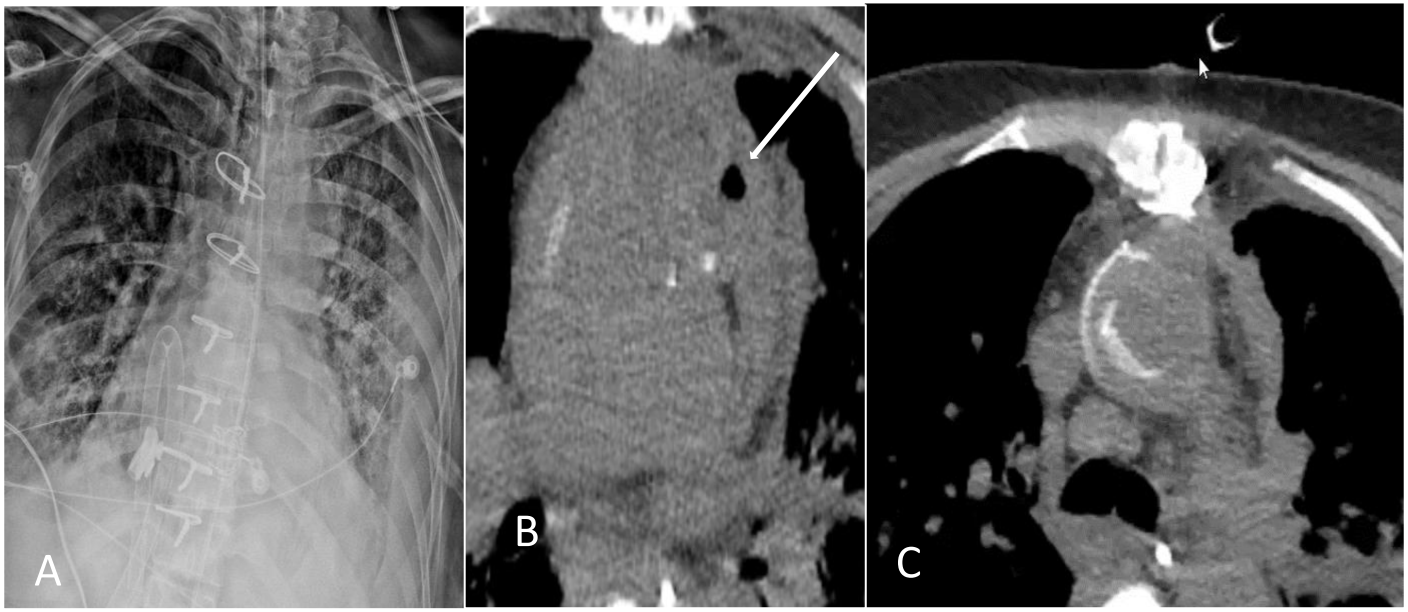

Supplemental Figure 2: Mediastinal infection. AP Chest radiograph (A) in a 55-year-old female with ascending aortic dissection status post Bentall procedure and placed on VA-ECMO with a tandem heart demonstrating a central VA ECMO cannula (white arrow) entering the RA and extends into the IVC. Chest CT (B, C) demonstrates a foci of air (white arrow) within the fluid collection adjacent to the venous ECMO cannula and Bentall graft (arrowhead) can be seen in the anterior mediastinum. Air within the collection is indicative of infection except in the immediate post operative period.

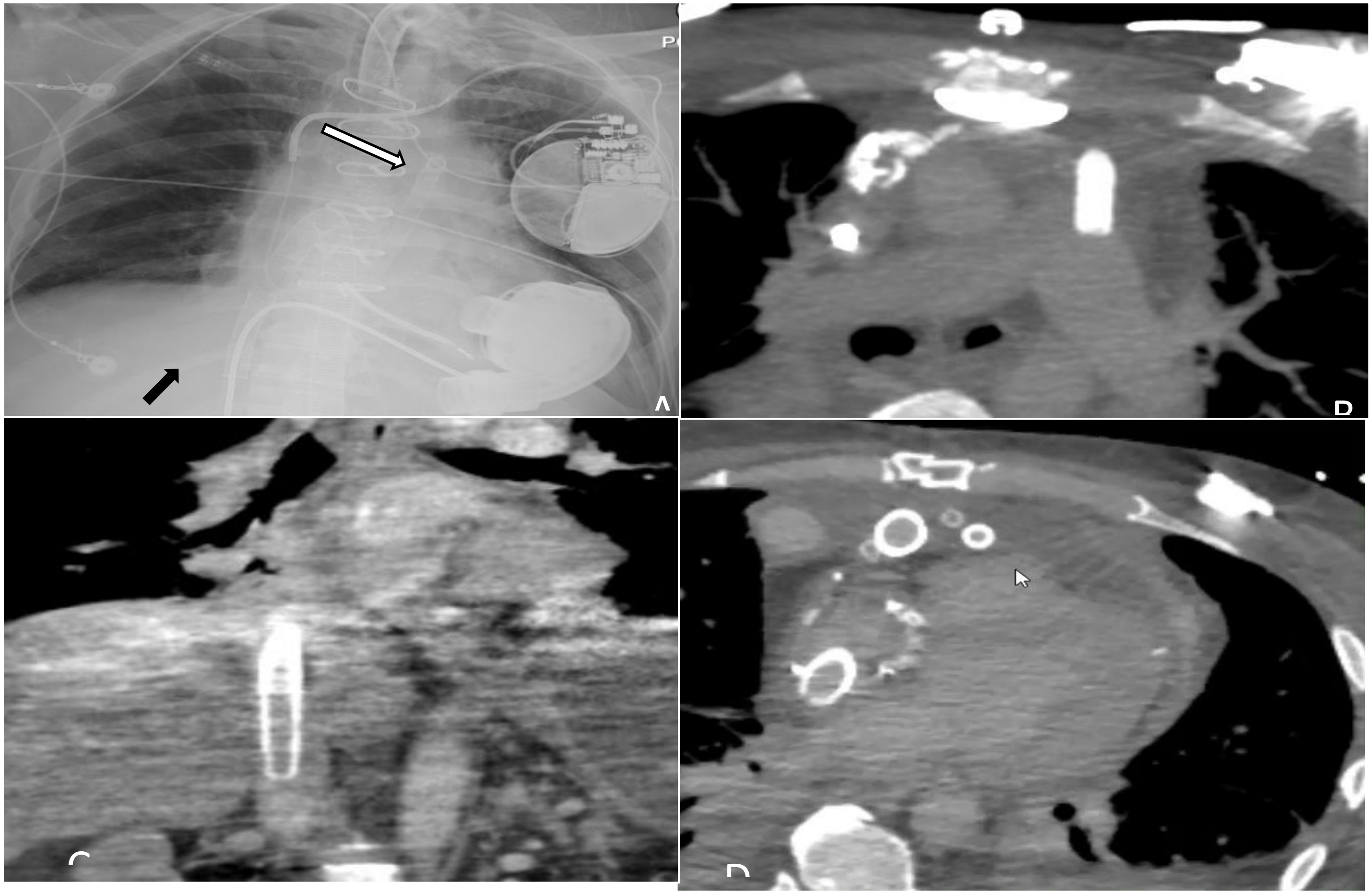

Supplemental Figure 3: Normal appearance of mediastinal cannula in a patient with Tandem heart. 58-year-old female with dilated cardiomyopathy and tricuspid valve repair status post HeartMate 2 implantation and central RVAD using a Tandemheart. Frontal chest radiograph (A) and axial (B,D) and coronal chest CT (C) shows ideal positioning of the inflow cannula within the IVC (black

arrows) and outflow cannula within the main pulmonary artery (white arrows). Fluid surrounding these central cannula is normal in the immediate postoperative period.

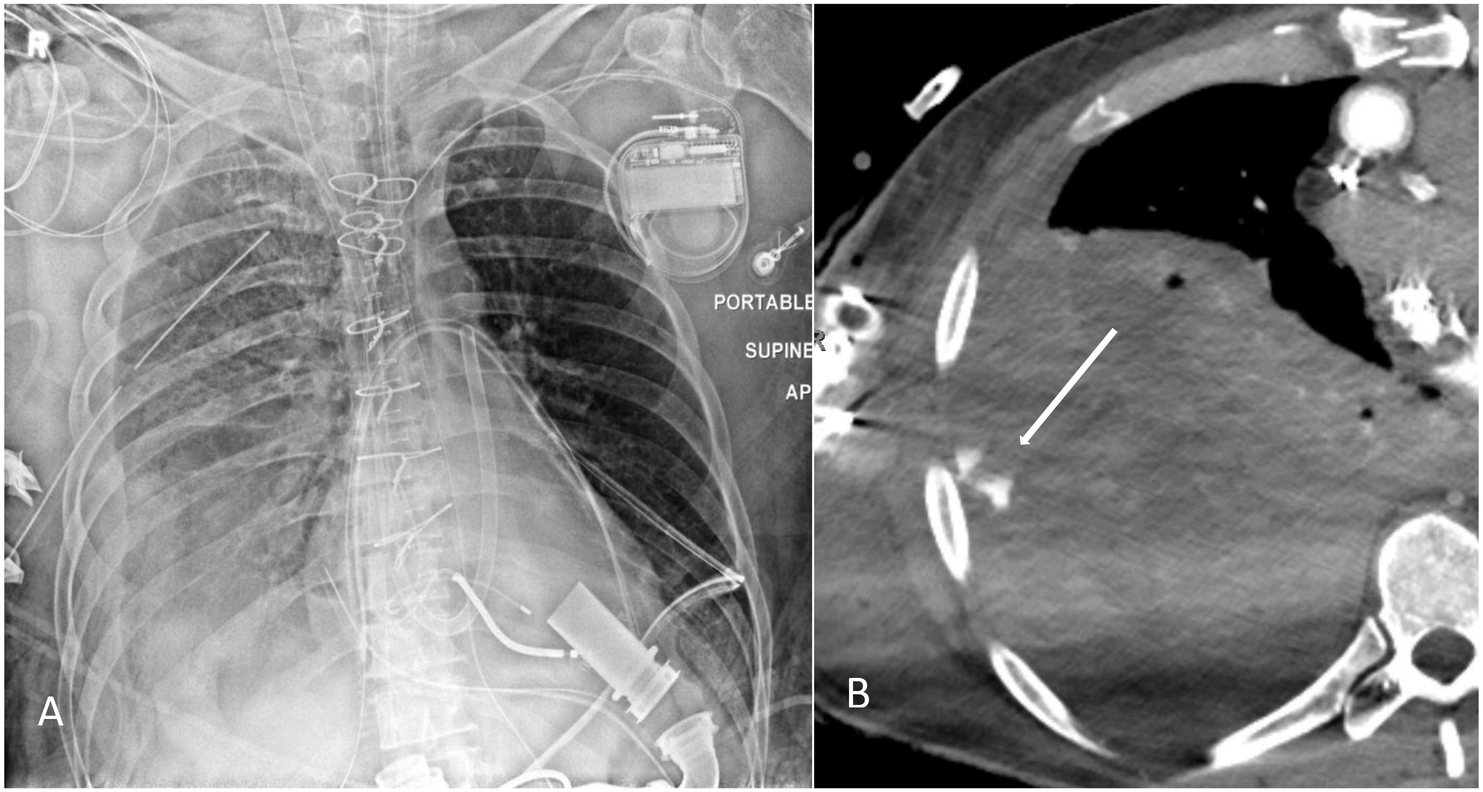

Supplementary Figure 4: Active bleeding. AP Chest radiograph (A) showing large right pleural effusion in a patient on biventricular support with Heartmate II and Protek-Duo. Axial contrast enhanced CT chest (B) demonstrates active contrast extravasation into the chest wall and pleural space (white arrow) with a large hemothorax.

Video Legends

ESM Video 1: Axial contrast enhanced CT of the chest abdomen and pelvis demonstrates low position of the intraoartic ballon pump. There is hypoperfusion and pneumatosis of the cecum.

ESM Video 2: Axial ECG gated CT of the thorax demonstrates malposition of the Impella pump. There is perforation of the left ventricle lateral wall. Pigtail portion of the Impella pump is present in the pericardium.
